# Psychometric properties of BREALD-30 for assessing adolescents’ oral health literacy

**DOI:** 10.11606/S1518-8787.2019053000999

**Published:** 2019-08-12

**Authors:** Larissa Chaves Morais de Lima, Érick Tássio Barbosa Neves, Laio da Costa Dutra, Ramon Targino Firmino, Luiza Jordânia Serafim de Araújo, Saul Martins Paiva, Fernanda Morais Ferreira, Ana Flávia Granville-Garcia

**Affiliations:** I Universidade Estadual da Paraíba. Programa de Pós-Graduação em Odontologia. Campina Grande, PB, Brasil; II Universidade Federal de Minas Gerais. Faculdade de Odontologia. Programa de Pós-Graduação em Odontologia. Belo Horizonte, MG, Brasil; III Universidade Estadual da Paraíba. Departamento de Odontologia. Campina Grande, PB, Brasil; IV Universidade Federal de Minas Gerais. Faculdade de Odontologia. Departamento de Odontopediatria e Ortodontia. Belo Horizonte, MG, Brasil

**Keywords:** Adolescent, Health Literacy, Oral Health, Validation Studies

## Abstract

**OBJECTIVE:**

To evaluate the psychometric properties of the Brazilian Portuguese version of the Rapid Estimate of Adult Literacy in Dentistry (BREALD-30) administered to adolescents.

**METHODS:**

The study included 750 adolescents: 375 aged 12 years and 375 aged 15–19 years, attending public and private schools in Campina Grande, state of Paraíba, Brazil, in 2017. Reliability was measured based on internal consistency and test-retest reliability. Convergent validity was measured based on correlations between BREALD-30 and Functional Literacy Indicator scores. Divergent validity was measured by comparing BREALD-30 scores with sociodemographic variables. For predictive validity, the association between BREALD-30 scores and the presence of cavitated carious lesions was tested using a multiple logistic regression model. All statistical tests were performed with a significance level of 5%.

**RESULTS:**

BREALD-30 showed good internal consistency for the 12 year olds and 15 to19 year olds (Cronbach’s alpha = 0.871 and 0.834, respectively) and good test-retest reliability [intraclass correlation coefficient (ICC) = 0.898 and 0.974; kappa = 0.804 and 0.808, respectively]. Moreover, item-total correlation was satisfactory for all items. BREALD-30 had convergent validity with the Functional Literacy Indicator for 12 year olds (rs = 0.558, p < 0.001) and for 15 to 19 year olds (rs = 0.652, p < 0.001). Participants with higher oral health literacy levels who attended private schools (p < 0.001), belonged to economic classes A and B2 (p < 0.001), and who had parents with higher education levels (p < 0.001) were included, indicating the divergent validity of the BREALD-30. Participants with lower BREALD-30 scores were more likely to have cavitated carious lesions [12 year olds: odds ratio (OR) = 2.37; 95% confidence interval (95%CI): 1.48–3.80; 15 to 19 year olds: OR = 1.96; 95%CI 1.24–3.11].

**CONCLUSIONS:**

BREALD-30 shows satisfactory psychometric properties for use on Brazilian adolescents and can be applied as a fast, simple, and reliable measure of oral health literacy.

## INTRODUCTION

Functional literacy comprises a set of skills, such as reading, writing, basic mathematic operations, speech, and the ability to perform particular tasks^1^. In dentistry, oral health literacy (OHL) is the ability to understand information regarding dental services, as well as the prevention, control, and treatment of oral problems^2,3^. This is a relevant concept in the realm of public health^4^, as adequate OHL leads to reduced risk behaviors, avoids greater costs to the public health system, and it improves health indicators^5,6^.

A high level of OHL improves the self-perception regarding oral health, the understanding of medical prescriptions, and the practice of proper oral hygiene^7^of individuals. In contrast, low OHL levels affect dentist–patient communication and hinder shared decision-making, which is a determinant of adherence to treatment, and consequently, the treatment outcome^4^. The literature shows that young people with a low level of health literacy are more prone to inadequate and risky behaviors, such as drug use, unsafe sex^10,11^, violent behavior^12^, medication errors, poor oral hygiene, and underuse of health prevention services^11,13^.

The Rapid Estimate of Adult Literacy in Dentistry (REALD-30) is one of the main assessment tools for screening low OHL levels in adults based on the recognition of words, and, according to a recent systematic review^14^, it is the most widely used assessment tool for this purpose worldwide. REALD-30 has been validated for use in the Brazilian population, and the version translated into Brazilian Portuguese has been designated BREALD-30^15^.

Only five assessment tools described in the literature measure health literacy in adolescents (participants aged 9–19 years)^16^. Although most of these assessment tools show good internal consistency for this age group, a scarcity of valid tools for assessing the construct in adolescents still remains^3,16^.

It is therefore important to determine the psychometric properties of assessment tools to determine OHL in other age groups, as distinct age groups have different needs and language use. Adequate psychometric properties enable scientifically consistent results. Administering a valid and reliable assessment tool that measures this construct in adolescents will change the Brazilian oral health scenario, increase individual life expectancy and promote quality of life^16^.

The hypothesis tested in this study is that BREALD-30 is valid and reliable for adolescents. Therefore, the aim of our study was to evaluate the psychometric properties (validity and reliability) of BREALD-30 administered to Brazilian adolescents in two age groups: 12 years and 15–19 years.

## METHODS

### Ethical Considerations

This study was approved by the Human Research Ethics Committee of the Universidade Estadual da Paraíba (Certificate 55953516.2.1001.5187) in compliance with Resolution 466/2012 of the Brazilian National Board of Health and with the Declaration of Helsinki.

### Study Design

This was a validation study with an analytical and cross-sectional approach to evaluate the psychometric properties (reliability and validity) of BREALD-30 administered to adolescents.

### Participants

The study population was composed of adolescents from two age groups (12 years and 15–19 years) who attended 10 public and 17 private schools that were randomly selected by lottery in the Microsoft^®^ Excel software (Microsoft^®^ Office, Excel version 15.0, 2013) in the city of Campina Grande, Northeastern Brazil. These age groups were chosen because they represent index ages adopted by the World Health Organization (WHO)^17^ for studies involving oral health in adolescents. As BREALD-30 has 30 items, 300 individuals were selected for each age group. Considering a possible dropout rate of 20%, 75 participants were added to each age group, resulting in a total of 750 participants^18^.

### Eligibility Criteria

Male and female adolescents aged 12 years and 15–19 years enrolled at public and private schools in Campina Grande and present on the day of data collection were included in the study. The exclusion criteria were inability to read or write^19^, inability to speak^19^, native language other than Portuguese, vision or hearing impairment, use of an orthodontic appliance, and visible intoxication by alcohol or drugs, which impeded participation in the study^15^.

### Training and Calibration

Before data collection, two researchers were trained and calibrated to administer the BREALD-30 following the methods proposed by the authors, who validated the questionnaire for adults^3^. Intraclass correlation coefficients (ICC) for agreement on total BREALD-30 scores between the raters and supervisor (a researcher with ample theoretical and practical experience in studies involving BREALD-30) and intra-rater agreement were ≥ 0.97 [95% confidence interval (95%CI): 0.921–0.998]. An ICC > 0.75 indicates an excellent level of agreement^20^. When agreement was calculated for each word of the BREALD-30, inter- and intra-rater kappa coefficients were ≥ 0.87 (p < 0.05).

To evaluate dental caries used in the predictive validity analysis based on the diagnostic index proposed by Nyvad^21^, the calibration of both raters followed the method proposed by Peres et al.^21^, and kappa values were ≥ 0.88 (p < 0.05). Before data collection, we conducted a pilot study involving 30 adolescents to improve the study plan.

### Collection of non-Clinical Data

Parents/caregivers answered a semi-structured questionnaire with objective questions addressing sociodemographic characteristics (age, income, marital status, schooling, number of residents at home, type of housing), as well as the Brazilian Economic Classification Criteria questionnaire, which is used to classify the population based on the number of consumer goods at the moment of the survey^23^. The economic classes (from highest to lowest) are A1, A2, B1, B2, C, D, and E. Family income was also collected in the number of Brazilian minimum wages and was used as a continuous variable. Data on the adolescents were also collected (sex, age, race, birth order, presence/absence of a private health insurance plan). Next, the BREALD-30 and Functional Literacy Indicator (FLI) were administered to each adolescent individually in interview form in a reserved room at the school.

BREALD-30 is the Brazilian Portuguese version of a screening tool that identifies low OHL in respondents, which is measured based on the recognition of 30 dental terms organized in increasing order of pronunciation difficulty and read out loud. Each word is scored 1 (correct pronunciation) or 0 (incorrect pronunciation). The total ranges 0–30 points, with lower scores denoting a lower OHL level^3^. In this study, the total BREALD-30 score was categorized in terciles^24^ to classify the OHL level.

The Functional Literacy Indicator (FLI) was used to analyze the convergent validity. FLI distinguishes two forms of literacy (words and numbers) that relate to reading skills and mathematical knowledge in social practices and to texts from daily living. The psychometric properties of FLI have been proven adequate^25^. To determine the level of functional literacy, the adolescents answered to 10 questions of FLI, following the methods adopted by other researchers^15,26^. The questions address all levels of functional literacy (illiteracy, and rudimentary, basic, and full knowledge) and are arranged in increasing order of difficulty. The answers are categorized and receive a score of 1 when correct or 0 when incorrect. The final score ranges 0–10, with higher scores denoting a higher level of functional literacy.

### Clinical Data Collection

After questionnaire administration and supervised toothbrushing, clinical data on dental caries were collected for the subsequent predictive validity analysis. In a reserved room, the adolescents were examined individually while seated in front of the rater, who wore personal protective equipment and a head lamp (Petzl Zoom headlamp, Petzl America, Clearfield, UT, USA). The intraoral examinations were performed with the aid of previously sterilized mouth mirrors (PRISMA, São Paulo, SP, Brazil) and WHO-621 periodontal probes (Trinity, Campo Mourão, PA, Brazil), as well as sterile gauze to dry the teeth^20^.

The index proposed by Nyvad^21^ was used for dental caries diagnosis. The index was developed to reflect the entire continuum of caries lesion development, ranging from clinically sound surfaces, through noncavitated and microcavitated enamel caries lesions, to frank cavitation into the dentin, and they have been successfully applied in clinical caries management and research.

### Statistical Analysis

For the reliability analysis, internal consistency was evaluated using Cronbach’s alpha (α) coefficient and the item-total correlation of BREALD-30. Test-retest reliability was measured using the kappa coefficient for the scores on each word during the initial administration as compared with the scores during a second administration to approximately 20% of the sample (n = 80) after a 14-day period, following the method proposed by other researchers^18^. ICC was also used to compare the total BREALD-30 scores on both occasions.

Construct validity was measured based on convergent, divergent, and predictive validity. Convergent validity was determined based on the correlation between the BREALD-30 and FLI scores, with the hypothesis that individuals with higher functional literacy levels, measured using the FLI, would have higher BREALD-30 scores. Spearman’s correlation coefficient was calculated for this analysis and the BREALD-30 and FLI variables showed non-normal distribution. For the divergent and predictive validity analyses, the total BREALD-30 scores were categorized as first, second, and third terciles based on the distribution of the adolescents’ responses. Using terciles as cut-off points, these categories corresponded to the respective scores of 0–16, 17–20, and 21–30 points (12-year-old group) and 0–18, 19–22, and 23–30 points (15–19-year-old group). Divergent validity was evaluated by comparing the BREALD-30 scores among the sociodemographic variables using the chi-square test and the chi-square test for linear trend. Predictive validity was evaluated by correlating the BREALD-30 score with the presence/absence of cavitated caries lesions (codes 3 and 6 of Nyvad^21^). The hypothesis was that adolescents with low OHL would have a more severe caries experience.

Multiple binary logistic regression models were created to test whether these associations were maintained after adjusting for possible confounding variables. The sociodemographic variables (12 years old: sex, race, parent/caregiver’s marital status, family income, absence/presence of a private health insurance plan, mother’s level of schooling; 15–19 years old: sex, race, type of school, absence/presence of a private health insurance plan, economic class, birth order, mother’s level of schooling, father’s level of schooling) obtained p < 0.20 in the bivariate analysis and were incorporated into the multivariate models. Using the stepwise backward regression method, the variables with the best fit (12 years old: household income, mother’s level of schooling, BREALD-30 score; 15–19 years old: household income, presence of private health insurance plan, BREALD-30 score) remained in the final model.

All statistical tests were performed using the Statistical Package for Social Sciences (SPSS for Windows, version 22.0, IBM Corp., Armonk, NY, USA).

## RESULTS

Regarding the sociodemographic characterization of the sample, 56.9% of the 12 year olds were girls, 67% considered themselves non-white, 40.7% were the oldest child, 69.4% had no private health insurance plan, and 39% belonged to economic class C. Among the 15–19 year olds, 58.4% were girls, 73% considered themselves non-white, 41.6% were the oldest child, 62.8% did not have a private health insurance plan, and 37% belonged to economic class B.

The mean BREALD-30 score was 18.5 (SD = 5.4) (median: 19) among the 12 year olds and 20.85 (SD = 4.7) (median: 21) among the 15–19 year olds. BREALD-30 showed good internal consistency in both groups [12 years old: Cronbach’s α = 0.871 (range: 0.865–0.871)]; 15–19 years old: α = 0.834 (range: 0.826–0.834]). Regarding the item-total correlations, all coefficients were around 0.30 in both groups (Table 1).

**Table 1:** Coefficients of internal consistency (Cronbach’s alpha), item-total correlations, and Cronbach’s alpha if the item was excluded, for the BREALD-30 administered to adolescents aged 12 years and 15–19 years.

Variable	12-year-old group		15–19-year-old group	
	
	Corrected item-total correlation	Cronbach’s alpha if item was excluded	Corrected item-total correlation	Cronbach’s alpha if item was excluded
Sugar	0.334	0.870	0.221	0.834
Denture	0.410	0.868	0.263	0.832
Smoker	0.411	0.867	0.286	0.832
Enamel	0.413	0.868	0.231	0.833
Dentition	0.464	0.865	0.403	0.827
Erosion	0.444	0.866	0.432	0.826
Genetics	0.479	0.866	0.310	0.831
Incipient	0.456	0.866	0.362	0.828
Gums	0.462	0.866	0.405	0.828
Restoration	0.411	0.867	0.266	0.831
Biopsy	0.458	0.866	0.432	0.826
Mouth wash	0.488	0.865	0.414	0.826
Bruxism	0.448	0.866	0.373	0.828
Brushing	0.472	0.865	0.382	0.828
Hemorrhage	0.482	0.865	0.420	0.827
Radiography	0.432	0.867	0.306	0.830
Film	0.464	0.866	0.408	0.828
Halitosis	0.373	0.868	0.306	0.830
Periodontal	0.352	0.869	0.301	0.831
Analgesia	0.248	0.871	0.315	0.830
Endodontic	0.160	0.871	0.298	0.830
Malocclusion	0.442	0.866	0.486	0.823
Abscess	0.423	0.867	0.482	0.823
Biofilm	0.421	0.867	0.247	0.832
Fistula	0.410	0.867	0.381	0.828
Hyperemia	0.397	0.867	0.378	0.828
Orthodontics	0.414	0.867	0.305	0.831
Temporomandibular	0.340	0.868	0.408	0.827
Hypoplasia	0.355	0.868	0.356	0.829
Apicectomy	0.376	0.868	0.415	0.826

Test-retest reliability was considered excellent in both groups [12 years old: ICC = 0.974 (95%CI 0.959–0.983), kappa coefficient = 0.804; 15–19 years old: ICC = 0.898 (95%CI 0.840–0.934), kappa coefficient = 0.808]. Convergent validity was determined using Spearman’s correlation test. Positive correlations were found between the BREALD-30 scores and functional literacy level as measured using FLI (12 years old: *r*_s_ = 0.558, p < 0.001; 15–19 years old: *r*_s_ = 0.652, p < 0.001).

In the analysis of divergent validity between OHL and the sociodemographic variables, 12 year olds with higher OHL levels were girls (p = 0.024) that attended private schools (p < 0.001), considered themselves white (p = 0.020), had a private health insurance plan (p < 0.001), belonged to economic class B (p < 0.001), and had parents with a college degree (p < 0.001) (Table 2). Among the 15–19 year olds, those with higher OHL levels attended private schools (p < 0.001), were the oldest child in the family (p < 0.001), belonged to economic classes A and B2 (p < 0.001), and had parents with a college degree (p < 0.001) (Table 3).

**Table 2:** Divergent validity analysis between OHL level (BREALD-30) and sociodemographic characteristics in the 12-year-old group. (n = 375)

Variable	Oral health literacy			

	1^st^ tercile	2^nd^ tercile	3^rd^ tercile	p
		
	(BREALD-30: 0–16)	(BREALD-30:17–20)	(BREALD-30:21–30)	
		
	n (%)	n (%)	n (%)	
Sex				0.024^a^
Male	53 (32.7)	59 (36.4)	50 (30.9)	
Female	68 (31.8)	54 (25.2)	92 (43.0)	
Race				0.020^a^
White	28 (22.8)	44 (35.8)	51 (41.5)	
Non-white	93 (36.9)	69 (27.4)	90 (35.7)	
Type of school				< 0.001^a^
Private	31 (16.5)	49 (26.1)	108 (57.4)	
Public	90 (47.9)	64 (34.0)	34 (18.1)	
Private health insurance				< 0.001^a^
No	96 (36.8)	86 (33.0)	79 (30.3)	
Yes	25 (21.7)	27 (23.5)	63 (54.8)	
Birth order				0.07^a^
Youngest	56 (46.3)	39 (34.5)	52 (36.6)	
Middle	26 (21.5)	26 (23.0)	24 (16.9)	
Oldest	39 (32.2)	48 (42.5)	66 (46.3)	
Economic class				< 0.001^b^
E	30 (50.0)	22 (36.7)	8 (13.3)	
D	41 (45.1)	28 (30.8)	22 (24.2)	
C	19 (32.2)	22 (37.3)	18 (30.5)	
B2	20 (21.1)	27 (28.4)	48 (50.5)	
B1	5 (13.2)	7 (18.4)	26 (68.4)	
A	4 (12.9)	7 (28.1)	20 (23.4)	
Mother’s schooling				< 0.001^b^
Illiterate or some primary school	24 (49.0)	13 (26.5)	12 (24.5)	
Primary school or some primary school	37 (50.7)	27 (37.0)	9 (12.3)	
Primary school or some high school	22 (30.6)	26 (36.1)	24 (33.3)	
High school or some college	25 (21.2)	32 (27.1)	61 (51.7)	
College degree	12 (19.0)	15 (23.8)	36 (57.1)	
Father’s schooling				< 0.001^b^
Illiterate or some primary school	31 (48.4)	18 (28.1)	15 (23.4)	
Primary school or some primary school	30 (37.5)	30 (37.5)	20 (25.0)	
Primary school or some high school	20 (33.3)	18 (30.0)	22 (36.7)	
High school or some college	31 (27.2)	34 (29.8)	49 (43.0)	
College degree	3 (6.5)	10 (21.7)	33 (71.7)	

^a^ Chi-square test*.*

^b^ Linear-by-linear association chi-square test*.*

**Table 3:** Divergent validity analysis between the OHL level (BREALD-30) and sociodemographic characteristics in the 15–19-year-old group. (n = 375)

Variable	Oral Health Literacy			

	1^st^ tercile	2^nd^ tercile	3^rd^ tercile	p
		
	(BREALD-30: 0-18)	(BREALD-30: 19-22)	(BREALD-30: 23-30)	
		
	n (%)	n (%)	n (%)	
Sex				0.152^a^
Male	35 (22.9)	48 (31.4)	70 (45.8)	
Female	61 (28.4)	77 (35.8)	77 (35.8)	
Race				0.094^a^
White	17 (17.7)	36 (37.5)	43 (44.8)	
Non-white	79 (29.0)	89 (32.7)	104 (38.2)	
Type of school				< 0.001^a^
Private	25 (13.8)	58 (32.0)	98 (54.1)	
Public	71 (38.2)	67 (35.5)	49 (26.3)	
Private health insurance				0.096^a^
No	61 (26.4)	79 (34.2)	91 (39.4)	
Yes	35 (25.5)	46 (33.6)	56 (40.9)	
Birth order				< 0.001^b^
Youngest	32 (25.6)	45 (36.0)	48 (38.4)	
Middle	34 (37.8)	23 (25.6)	33 (36.7)	
Oldest	30 (19.6)	57 (37.3)	66 (43.1)	
Economic class				< 0.001^b^
E	23 (44.2)	19 (36.5)	10 (19.2)	
D	19 (31.1)	17 (27.9)	25 (41.0)	
C	24 (29.6)	31 (38.3)	26 (32.1)	
B2	20 (20.2)	26 (26.3)	53 (53.5)	
B1	6 (16.2)	17 (45.9)	14 (37.8)	
A	4 (10.5)	15 (39.5)	19 (50.0)	
Mother’s schooling				< 0.001^b^
Illiterate or some primary school I	23 (39.7)	19 (32.8)	16 (27.6)	
Primary school or some primary school	22 (31.4)	27 (38.6)	21 (30.0)	
Primary school or some high school	22 (36.1)	22 (36.1)	17 (27.9)	
High school or some college	22 (19.1)	37 (32.2)	56 (48.7)	
College degree	7 (11.1)	19 (30.2)	37 (58.7)	
Father’s schooling				< 0.001^b^
Illiterate or some primary school	29 (36.7)	28 (35.4)	22 (27.8)	
Primary school or some primary school	30 (37.0)	25 (30.9)	26 (32.1)	
Primary school or some high school	16 (23.9)	23 (34.3)	28 (41.8)	
High school or some college	15(16.7)	27 (30.0)	48 (53.3)	
College degree	5(10.2)	21 (42.9)	23 (46.9)	

Values other than 375 are at isolated losses.

^a^ Chi-square test.

^b^ Linear-by-linear association chi-square test.

Regarding predictive validity, lower BREALD-30 scores (lower OHL) were associated with greater prevalence of cavitated carious lesions (p < 0.001). This association was maintained after adjustment for possible confounding variables in the multiple logistic regression models. In the model adjusted for income and mother’s level of schooling, the 12 year olds with a low OHL level (BREALD-30 score: 0–16) had 2.37-fold greater odds [odds ratio (OR) = 2.37; 95%CI 1.48–3.80] of having cavitated carious lesions compared with those with a higher OHL level (Table 4). After adjustments for income and the presence of a private health insurance plan, the 15–19 year olds with a low OHL level (BREALD-30 score: 0–18) had approximately 2-fold greater odds (OR = 1.96; 95%CI 1.24–3.11) of having cavitated carious lesions (Table 4).

**Table 4:** Multiple logistic regression model for association between OHL (BREALD-30) and health outcome (presence of caries) in the 12-year-old group and in the 15–19-year-old group (predictive validity).

Age group	Predictor	Cavitated carious lesions	

		p	Adjusted OR (95%CI)
12 years	Household income (minimum wages)	0.064	0.85 (0.72–1.00)
	Mother’s schooling		
	Illiterate	0.016	2.57 (1.20–5.51)
	Primary or some primary education	0.020	2.35 (1.15–4.81)
	Primary or some high school	0.035	2.21 (1.16–4.60)
	High school or some college	0.290	1.44 (0.73–2.83)
	College degree (ref)		
	Oral Health Literacy		
	1^st^ tercile (BREALD-30: 0–16)	< 0.001	2.37 (1.48–3.80)
	2^nd^ tercile (BREALD-30: 17–20)	0.105	1.50 (0.92–2.25)
	3^rd^ tercile (BREALD-30: 21–30) (ref)		
19 years	Household Income (minimum wages)	0.002	0.77 (0.65–0.91)
	Private Health Insurance		
	Yes	0.040	0.66 (0.44–0.90)
	No (ref)		
	Oral Health Literacy		
	1^st^ tercile (BREALD-30: 0–18)	0.004	1.96 (1.24–3.11)
	2^nd^ tercile (BREALD-30: 19–22)	0.005	1.88 (1.21–2.93)
	3^rd^ tercile (BREALD-30: 23–30) (ref)		

BREALD-30: Rapid Estimate of Adult Literacy in Dentistry

## DISCUSSION

BREALD-30 had high Cronbach’s alpha coefficients for both the 12-year-old (α = 0.82) and 15–19-year-old (α = 0.87) groups, showing substantial internal consistency. These values are similar to those found in the BREALD-30 validation study for adults in Brazil (α = 0.87)^15^. Assessment tools with α ≥ 0.70 are considered acceptable^27^.

As to stability, Kappa coefficients were considered good in the 12-year-old and 15–19 year old groups (0.804 and 0.808, respectively)^28^. In all analyses, ICC was > 0.89 and within the range established by other researchers on this topic^29^.

In the analysis of the item-total correlations, items that correlate well with the total score have values near or higher than 0.30, whereas lower values indicate that an item may not be correlated with the total score of the scale and should be removed. In this study, the term “endodontic” had a coefficient of 0.16 in the 12 year old group and therefore Cronbach’s alpha for the total did not increase with the exclusion of this item^30^.

The mean BREALD-30 score was 18.5 for the 12-year-old group and 20.85 for the 15–19-year-old group. These scores are lower than that reported in the BREALD-30 validation study for adults (21.6)^15^, which may be explained by the fact that adults have more exposure to words related to oral health throughout life^31^. Moreover, adolescents are usually less concerned with health and oral health issues compared with adults, which can reduce their exposure to dental terms^16^.

The adaptation of BREALD-30 for adolescents is the first attempt to furnish a measure for evaluating the OHL in this age group in Brazil^14^. This is one of the few oral health-related assessment tools described in the literature that has undergone important validation steps such as translation and cross-cultural adaptation (conceptual, item, semantic, and operational equivalence) and that has shown satisfactory psychometric properties^15^.

BREALD-30 showed adequate convergent validity for both 12-year-old and 15–19 year old groups, which is in agreement with the findings of validation studies that also employed the FLI^15,26^; the correlation coefficients (*r*_s_) were 0.59 and 0.60, respectively. Spearman’s coefficients > 0.5 indicate moderate correlations, which are considered satisfactory for validation studies^18^.

Regarding divergent validity, girls and those who considered themselves white in the 12-year-old group had higher OHL levels, corroborating the literature, for which the possible association of these variables is clear^20,24,26^. In both age groups studied, most adolescents with a low OHL level were from less privileged economic classes and had parents with lower levels of schooling. Moreover, studying at a public school was associated with a lower OHL level in both groups (p < 0.001), which is in agreement with the findings of a previous study conducted in Brazil^26^. These data underscore the importance of measuring the OHL of the population before planning educational actions. Regarding other variables used to evaluate divergent validity, having private health insurance was associated with OHL among the 12 year olds, and being the oldest child in the family was associated with OHL among 15–19 year olds. The association between OHL and some of these variables reflect the greater degree of independence among older adolescents seeking information and services^15,31^.

Another interesting finding was that adolescents with lower OHL were significantly more likely to have cavitated carious lesions, even after controlling for possible confounding variables. Subjects with lower OHL may face more difficulties in understanding oral health instructions or may be less concerned with preventive measures, being more likely to have dental caries. Previous validation studies of REALD-30 have also reported a similar trend^2,14^.

Previous investigations only used non-clinical data to analyze predictive validity, such as self-perception of oral health^15,26^, toothbrushing frequency^24^, and last visit to the dentist^15^. Therefore, this study represents an advance in knowledge, as it investigated the association between the OHL level and a clinical variable (cavitated carious lesions) in adolescents.

An intrinsic flaw of BREALD-30 is that it only measures word recognition and does not test the understanding of what is read^6^. To avoid response bias, the questions related to socioeconomic condition were applied in the form of a coded self-administered questionnaire, guaranteeing the respondent’s anonymity. Nonetheless, a fast, simple, and reliable assessment tool enables health professionals to adapt their use of language to ensure better communication with patients. BREALD-30 has been successfully used in previous studies involving the adult population^3,15^.

In conclusion, BREALD-30 shows adequate psychometric properties for measuring OHL among adolescents who speak Brazilian Portuguese through the recognition of dental terms. Moreover, the findings show that lower OHL levels are associated with poorer clinical status (cavitated carious lesions). Considering Brazil’s size and cultural diversity, it will be interesting to observe in further studies whether the psychometric properties are maintained with adolescents from other regions.
